# A PXR-Mediated Negative Feedback Loop Attenuates the Expression of CYP3A in Response to the PXR Agonist Pregnenalone-16α-Carbonitrile

**DOI:** 10.1371/journal.pone.0016703

**Published:** 2011-02-02

**Authors:** Ian Bailey, G. Gordon Gibson, Kathryn Plant, Mark Graham, Nick Plant

**Affiliations:** 1 Faculty of Health and Medical Sciences, Centre for Toxicology, University of Surrey, Surrey, United Kingdom; 2 Safety Assessment, AstraZeneca R&D Charnwood, Leicester, United Kingdom; Institut de Génomique Fonctionnelle de Lyon, France

## Abstract

The nuclear receptor superfamily of ligand-activated transcription factors plays a central role in the regulation of cellular responses to chemical challenge. Nuclear receptors are activated by a wide range of both endogenous and exogenous chemicals, and their target genes include those involved in the metabolism and transport of the activating chemical. Such target gene activation, thus, acts to remove the stimulating xenobiotic or to maintain homeostatic levels of endogenous chemicals. Given the dual nature of this system it is important to understand how these two roles are balanced, such that xenobiotics are efficiently removed while not impacting negatively on homeostasis of endogenous chemicals. Using DNA microarray technology we have examined the transcriptome response of primary rat hepatocytes to two nuclear receptor ligands: Pregnenalone-16α-carbonitrile (PCN), a xenobiotic PXR agonist, and lithocholic acid, an endogenous mixed PXR/VDR/FXR agonist. We demonstrate that despite differences in the profile of activated nuclear receptors, transcriptome responses for these two ligands are broadly similar at lower concentrations, indicating a conserved general response. However, as concentrations of stimulating ligand rises, the transcriptome responses diverge, reflecting a need for specific responses to the two stimulating chemicals. Finally, we demonstrate a novel feed-back loop for PXR, whereby ligand-activation of PXR suppresses transcription of the PXR gene, acting to attenuate PXR protein expression levels at higher ligand concentrations. Through *in silico* simulation we demonstrate that this feed-back loop is an important factor to prevent hyperexpression of PXR target genes such as CYP3A and confirm these findings *in vitro*. This novel insight into the regulation of the PXR-mediated regulatory signal networks provides a potential mechanistic rationale for the robustness in steroid homeostasis within the cell.

## Introduction

Within biological systems there is a requirement for robustness, defined as the ability of that biological system to continue to carry out its fundamental tasks [1]. Perfect robustness within a network is unlikely to occur unless the network fulfils some very specific criteria [2], and indeed such perfect robustness is probably relatively rare in biological systems. However, the use of feed-back and feed-forward mechanisms allows systems to adapt to alterations in environmental conditions, thus achieving quasi-robustness. With respect to drug metabolism, the processes' of adsorption, distribution, metabolism and excretion (ADME) following chemical exposure are targeting at reducing the level of the challenging chemical back to the pre-challenge state, i.e. zero. This, in itself, is a relatively simple aim; however, as these same ADME processes' also control the fate of endogenous chemicals within the body, any alteration in ADME processes caused in response to challenge by external chemicals will potentially impact upon these endogenous processes, potentially leading to pathophysiology [3], or on other xenobiotics, potentially resulting in clinically relevant drug-drug interactions [4]. There is thus a need for balance within the ADME system, such that central body functions are not overtly impacted by xenobiotic-mediated alterations (quasi-robustness), yet the system is still able to respond effectively to xenobiotic challenge, or extreme fluctuations in endogenous chemical levels suggestive of pathophysiology (sensitivity). This balance is achieved through three, interconnected systems: First, promiscuity within the ligand specificity for transporters and drug metabolizing enzymes ensures that for any given chemical, whether it is endogenous or exogenous in origin, several complimentary systems can mediate the efficient removal of the stimulating chemical [5], [6], [7], [8], [9]. Second, activation of ADME pathways is closely coupled with the activation of protective mechanisms that remove toxic damage elicited by stimulating chemicals should it occur before the chemical can be safely removed [10], [11]. Third, impact of chemicals on ADME pathways are subject to complex feed-back and feed-forward loops at the nuclear receptor level, coordinating expression levels of both metabolic and transporter proteins with their requirement to handle fluctuating chemical levels [7], [12], [13], [14]. These latter networks have become an area of intense study, as their dysfunction may underlie some of the observed adverse effects observed following chemical exposures. In addition, manipulation of these networks represents an exciting novel therapeutic area for the treatment of endogenous chemical metabolism dysfunction [15], [16].

The pregnane X-receptor (PXR, NR1I2) is an important regulator of a number of target genes involved in the metabolism of xenobiotics, including both metabolic enzymes and drug transporters [17], [18]. It is activated by a wide range of xenobiotics [19], and is itself regulated by a number of other nuclear receptors [14], [20], [21], highlighting it as an important node in the regulatory signal network concerned with body responses to xenobiotics. In addition to this established role as a xenosensing nuclear receptor it is important to note that PXR target genes are also important in a number of physiological functions, including haem, bile and cholesterol synthesis [22]: It is therefore pertinent to ask how PXR-mediated effects on xenobiotic and endobiotic metabolism are balanced to achieve both the sensitivity to xenobiotic challenge, but allow robustness in endogenous steroid metabolism.

To study this, we have examined the target gene set activated by two nuclear receptor agonists, lithocholic acid (LCA) and pregnenalone-16α-cabonitrile (PCN). LCA is an endogenous bile acid, which acts as an agonist for PXR, the farnesoid X-receptor (FXR) and the vitamin D receptor (VDR), whereas PCN is an exogenous antiglucocorticoid, which acts solely as a PXR agonist. Using DNA microarray technology we examine the hypothesis that differential nuclear receptor regulation by the two chemicals allows an efficient response to the stimulating chemical, while minimising impact on endogenous steroid metabolism.

## Results

### Differential activation of classic target genes for PXR, VDR and FXR target gene expression by LCA and PCN in primary rat hepatocytes

**Figure 1:**
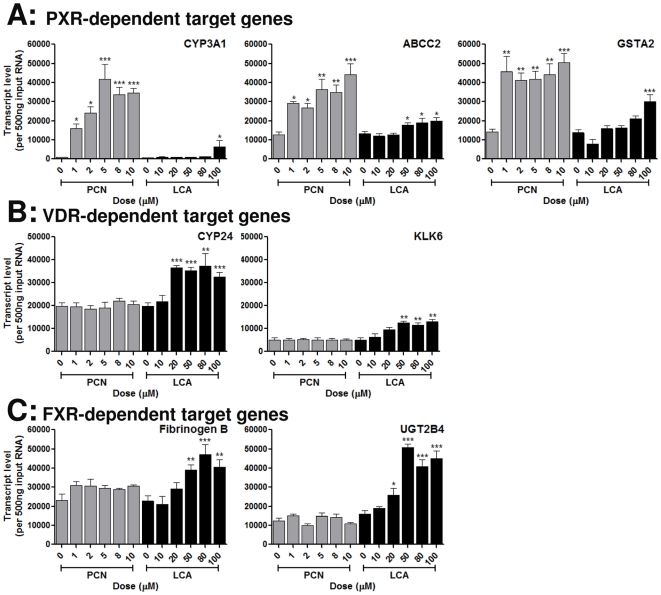
Induction of PXR-, FXR- and VDR-specific target genes by PCN and LCA. Quadruplicate cultures of primary rat hepatocytes were exposed to varying concentrations of PCN or LCA for 48 hours. RNA was extracted and target gene expression measured at the transcript level using TaqMan Q-PCR. Target genes represent those specifically activated by PXR (A), VDR (B) and FXR (C). Statistical analysis against vehicle control was determined via one-way ANOVA; * = p<0.01, ** = p<0.05 and *** = p<0.001.

Both PCN and LCA have been reported as PXR agonists [23], while LCA is also a known agonist for FXR [24] and VDR [25], with the relative EC50s for target gene activation suggesting an affinity of VDR>FXR>PXR. To confirm that this differential induction profile primary rat hepatocytes were grown in a sandwich cell culture system as described in Howe et al [26]; growth of hepatocytes between two layers of collagen permits correct polarisation of the cells and formation of functional bile cannaliculi, which may be important when studying the action of bile salts such as LCA. To determine a relative order of potency, we first examined the transcript levels of classical PXR, VDR and FXR target genes in response to 48 hours exposure to either LCA or PCN: For this purpose CYP3A1, GSTA2 and ABCC2 represent PXR target genes [23], CYP24 and KLK6 represent VDR target genes [25], while Fibrinogen β and UGT2B4 represent FXR-target genes [24]. As can be seen from Figure 1, the PXR target genes CYP3A1, GSTA2 and ABCC2 all demonstrate significant dose-dependent increases in transcript levels in response to both PCN and LCA, although with a generally lower potency with LCA (Figure 1a). In comparison, the VDR-target genes CYP24 and KLK6 are only activated by LCA (Figure 1b), as is the case for the FXR target genes Fibrinogen β and UGT2B4 (Figure 1c). The relative potency of LCA as agonist for PXR, FXR and VDR can be estimated through the EC50s of induction for their respective target genes (Table 1), and is consistent with the published literature, being VDR>FXR>>PXR.

**Table 1:** Relative potency for the induction of PXR, VDR and FXR target genes by PCN and LCA.

Target Gene	Activating NR	LCA EC50 (µM)	PCN EC50 (µM)
CYP3A1	PXR	>80	1.4±0.6
ABCC2	PXR	10±7	0.6±0.4
GSTA2	PXR	35±24	0.1±0.2
CYP24	VDR	5±4	N.D.
KLK6	VDR	11±5	N.D.
Fibrinogen β	FXR	14±8	N.D.
UGT2B4	FXR	18±15	N.D.

N.D.  =  Not induction observed. Quadruplicate cultures of primary rate hepatocytes were exposed to varying concentrations of PCN or LCA for 48 hours. RNA was extracted and target gene expression measured at the transcript level using TaqMan Q-PCR. Dose response curves were fitted using GraphPad Prism (v5) and EC50 values derived. N.D.  =  not determined as no dose response relationship evident.

### PCN and LCA activate differential nuclear receptor expression patterns

**Figure 2:**
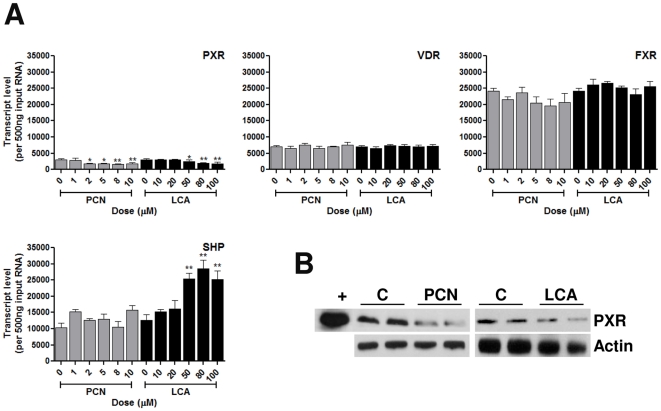
Nuclear receptor expression in response to PCN and LCA. Quadruplicate cultures of primary rat hepatocytes were exposed to varying concentrations of PCN or LCA for 48 hours. RNA and protein were extracted and nuclear receptor gene expression measured at the transcript (A) or protein (B) level using TaqMan Q-PCR or Western blotting, respectively. For protein levels, cells were exposed to either vehicle only (0.1%DMSO; C), 10 µM PCN or 100 µM LCA as indicated. Antibody fidelity was confirmed a TNT-expressed PXR protein (Lane ‘+’). Statistical analysis against vehicle control was determined via one-way ANOVA; * = p<0.01, ** = p<0.05 and *** = p<0.001.

One level of co-ordination of cellular responses to chemical challenge is the control of nuclear receptor transcription and/or activity by other nuclear receptors. To examine this, we measured PCN and LCA-mediated effects on nuclear receptor expression levels within primary rat hepatocytes. As can be seen from Figure 2, both PCN and LCA caused a dose-dependent suppression of PXR transcript levels (Figure 2a); in addition, this suppression was confirmed at the protein level, with PCN eliciting a marked decrease in PXR protein levels, and LCA exposure eliciting a smaller decrease (Figure 2b). In contrast, the expression of VDR and FXR appeared unaffected by either PCN or LCA exposure. We also examined the expression of the small-heterodimer partner (SHP), which can heterodimerise with activated receptors and prevent transactivation of DNA as it lacks a DNA binding domain [27]. SHP expression was unaffected by the PXR agonist PCN, but was significantly increased following exposure of rat hepatocytes to LCA (EC50  = 12.7±6.5 µM), consistent with its known role in response to bile acids [28].

### PCN- and LCA-mediated transcriptome changes

**Figure 3:**
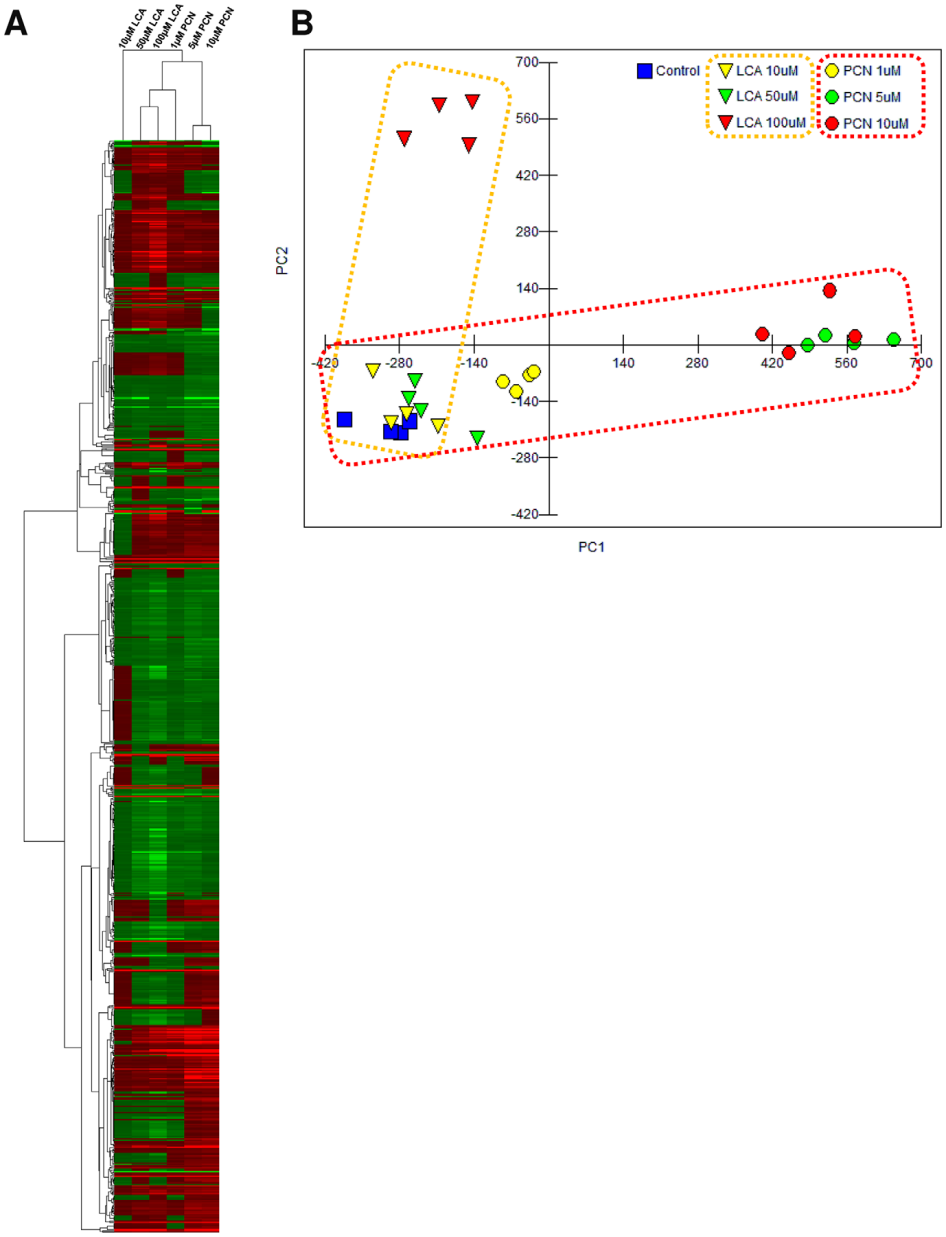
PCN and LCA activate divergent transcriptome profiles in a dose-dependent manner. Quadruplicate cultures of primary rat hepatocytes were exposed to PCN or LCA at the indicated concentrations for 48 hours. RNA was extracted and gene expression monitored using Affymetrix rat 230 v2 microarrays. Statistically significant transcript changes were identified by SAM analysis, and the analysed by PCA using MVSP.

Following the demonstration that both PCN and LCA activate classical target genes for PXR (PCN and LCA), FXR (LCA only) and VDR (LCA only), we next examined the total transcriptome response to these agents. Exposure of sandwich cultured hepatocytes to varying concentrations of either LCA or PCN for 48 hours resulted in a dose-dependent increase in the total number of genes activated, with similar total numbers of genes affected by both chemicals (Table 2). To further examine the response to either agent, and detect the major variables between the two datasets, multivariate statistical analysis was undertaken. Initially, heirachical cluster analysis using centroid linkage and an uncentered correlation metric was performed, producing the heatmap seen in Figure 3a. The heatmap demonstrates a clustering of higher dose (5 and 10 µM) PCN-treated hepatocytes, with a second cluster containing the low dose (1 µM) PCN, plus the higher dose (50 and 100 µM) LCA samples: This latter cluster presumably reflects the similar PXR-mediated activation of target gene sets by these treatments, and is consistent with the notion of LCA as a lower potency agonist than PCN.

**Table 2:** Gene expression changes elicited by PCN and LCA in primary rat hepatocytes.

Drug	Dose(**µ**M)	FDR(%)	Total genes altered	Gene Expression Increased	Gene Expression Decreased	Predicted False Positives
PCN	1	10	41	23	18	4
	5	9	147	33	114	13
	10	6	443	255	188	26
LCA	10	31	10	0	10	3
	50	3	253	55	198	8
	100	1	356	137	219	4

Quadruplicate cultures of primary rate hepatocytes were exposed to PCN or LCA at the indicated concentrations for 48 hours. RNA was extracted and gene expression monitored using Affymetrix rat 230 v2 microarrays. Statistical Analysis of Microarrays (SAM) analysis was undertaken to identify statistically significant gene expression changes (Dose vs medium). False Discovery Rate (FDR) was manually set to provide sufficient gene changes, while limiting the number of predicted false positives. Numbers of genes altered under each condition are presented for the stated FDR, along with the predicted number of false positives within each group.

**Figure 4:**
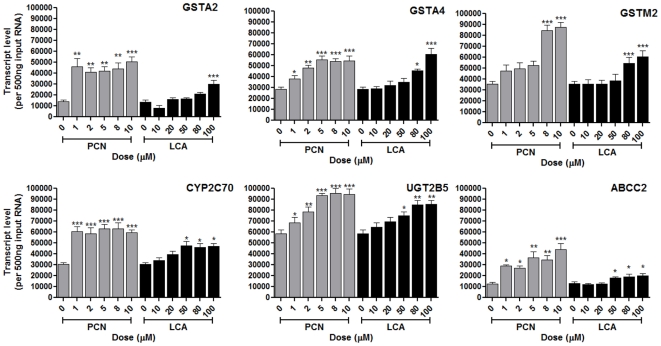
Transcript expression levels of PC1 eigengenes. Quadruplicate cultures of primary rat hepatocytes were exposed to varying concentrations of PCN or LCA for 48 hours. RNA was extracted and transcript levels for PC1 eigengenes determined using TaqMan Q-PCR. Statistical analysis against vehicle control was determined via one-way ANOVA; * = p<0.01, ** = p<0.05 and *** = p<0.001.

Next, principle component analysis (PCA) was used to examine the drivers for variability within the dataset. Figure 3b demonstrates that whereas the initial trajectories of the plots are the same, being primarily described by principal component 1, as the dose increases the trajectories separate along principal component 2. To further examine the divergence of dose response trajectories for PCN and LCA, the PCA eigenvectors associated with both principle components were analysed. PC1, which accounts for 38% of the total variation, has twenty eigengenes that drive the variability along this axis (Table 3). In general, these eigengenes are involved in ADME processes, either being metabolic enzymes or transport proteins, and the majority of these have their zexpression increased by both LCA and PCN; a selection of these eigengenes were analysed by Q-PCR, and the results are presented in Figure 4, demonstrating that these eigengenes are altered by both PCN and LCA exposures.

**Table 3:** Eigengenes for PCA of LCA and PCN-treated hepatocytes.

Affy ID	Gene Name	PC1	PC2
1367655_at	thymosin, beta 10	0.534	
1370952_at	GSTM2	0.297	
1368180_s_at	GSTA3	0.236	
1394109_at	thrombospondin 2	0.22	
1370698_at	UGT2B17	0.197	
1368397_at	UGT2B4	0.185	
1380865_at	Transcribed locus	0.156	
1387955_at	UGT2B5	0.145	
1367973_at	chemokine (C-C motif) ligand 2	0.143	−0.215
1387949_at	CYP2C22	0.122	
1372297_at	GSTA4	0.12	
1368718_at	ALDH1A4	0.114	
1368497_at	ABCC2	0.105	
1386886_at	CD164 molecule, sialomucin	−0.116	
1388506_at	Desmoplakin	−0.121	
1398846_at	eIF-5	−0.127	
1386902_at	voltage-dependent anion channel 3	−0.129	
1387777_at	integrin linked kinase	−0.137	
1388236_x_at	RT1-CE1	−0.137	
1370277_at	SLC25A3	−0.294	
1371237_a_at	metallothionein 1a		0.46
1388271_at	metallothionein 2A		0.344
1393236_at	RIO kinase 3		−0.189
1370281_at	FABP5		−0.196
1372687_at	cysteine-rich C-terminal 1		−0.357

Quadruplicate cultures of primary rate hepatocytes were exposed to varying concentrations of PCN or LCA for 48 hours. RNA was extracted and gene expression monitored using Affymetrix rat 230 v2 microarrays. Significantly altered transcript levels were determined via SAM analysis, then further analysed using PCA. Eigengenes describing the variation along principle component 1 (PC1) and 2 (PC2) are indicated, along with the eigenvalue for each eigengene against its primary component.

**Figure 5:**
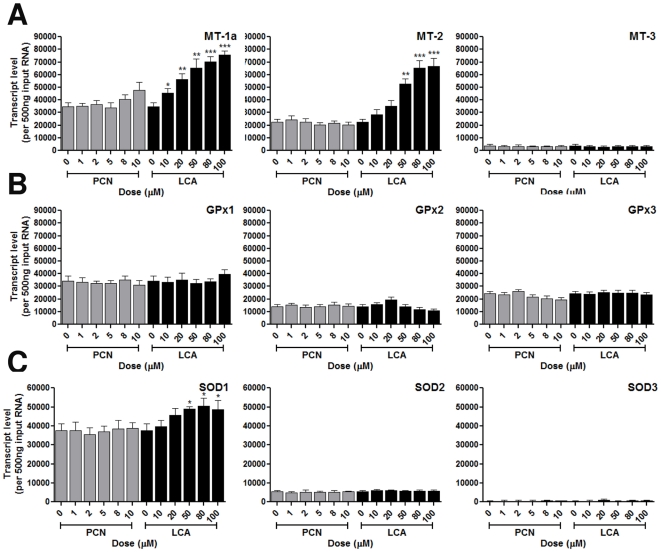
Transcript expression levels of PC2 eigengenes. Quadruplicate cultures of primary rat hepatocytes were exposed to varying concentrations of PCN or LCA for 48 hours. RNA was extracted and transcript levels for PC2 eigengenes (A), plus other oxidative stress response genes (B+C) determined using TaqMan Q-PCR. Statistical analysis against vehicle control was determined via one-way ANOVA; * = p<0.01, ** = p<0.05 and *** = p<0.001.

PC2, which accounts for 16% of the variability within the dataset, comprises 6 eigengenes (Table 3), including metallothionein 1a (MT-1) and metallothionein 2 (MT-2), with eigen values of 0.46 and 0.34 respectively. These changes were confirmed by Q-PCR, demonstrating that these eigengenes are altered by LCA, but not PCN, exposures (Figure 5a). The expression of MT-3 was also examined, demonstrating it is expressed at very low levels in the liver and unaffected by either PCN or LCA. Given the role of MTs in response to pro-oxidants [29], the expression of other oxidative stress response genes, the glutathione peroxiodase family (GPx; Figure 5b) and superoxide dismutase family (SOD; Figure 5c) were examined. No effect was seen in GPx expression levels, but LCA induced a significant dose-dependent increase in SOD1 transcript levels (Figure 5c). Thus, PC2 eigengenes are differentially affected by PCN and LCA, and reflect the antioxidant response elicited by high dose LCA exposure.

To examine the potential biological impact of these transcriptome alterations we undertook Gene Ontology (GO) analysis using the DAVID suite to identify significantly over-expressed biological pathways within the subset of transcripts regulated by PCN-alone, LCA-alone or by both chemicals. Table 4 shows that while the majority of gene expression changes are exclusive to either LCA or PCN, with only 9% of transcript changes being shared, at the level of GO biological pathway terms there is significant overlap (47%), suggestive of a highly similar biological response to both chemicals; this also demonstrates the importance of analysis at the pathway level, rather than just the individual transcript level, as more information on likely biological effects can be seen. GO classification terms are divided into five levels, with GO-level 1 representing terms of broadest meaning (e.g. physiological process), whereas GO-level 5 terms reflect much more precise definitions of a particular function (e.g. immune response). At the highest level of GO analysis (GO5), commonly regulated biological pathways, included those associated with cellular and protein metabolism, which is consistent with the observed joint inductions of metabolic enzymes, demonstrated in Figures 1 and 4. For LCA, there was a specific over-representation of pathways for nitrogen and amine catabolism, consistent with the response to raised secondary bile acid levels, as well as an over-representation of genes in pathways associated with apoptosis; this latter phenomenon probably represents the divergence of LCA along PC2 in the PCA analysis and shows the response to low-grade cholestatic insult.

**Table 4:** LCA and PCN activate different target genes, but markedly overlapping GO biological processes.

Direction	Gene Expression			GO Biological Process		
of Regulation	PCNAlone	LCAAlone	Both	PCNAlone	LCAAlone	Both
UP	272	150	24	13	6	14
DOWN	219	272	69	7	9	18

Quadruplicate cultures of primary rate hepatocytes were exposed to PCN or LCA for 48 hours. RNA was extracted and gene expression monitored using Affymetrix rat 230 v2 microarrays. Gene expression number relates to those identified as significantly altered via SAM analysis; functional annotation clustering was undertaken on these identified genes using DAVID, with GO biological process 3–5 as the search term.

### PXR autoregulation insulates endogenous metabolic processes from external stimuli

**Figure 6:**
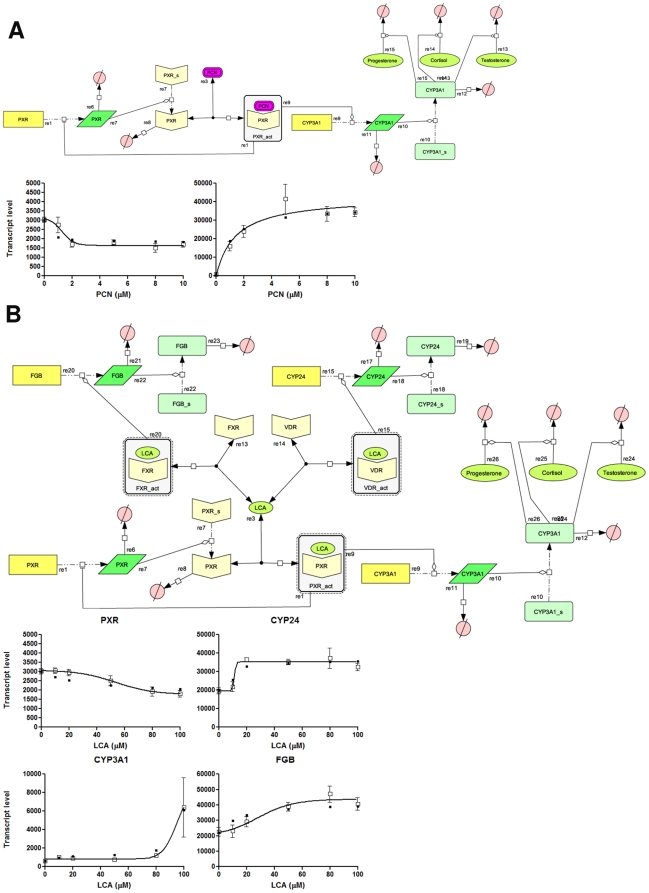
*In silico* models of PCN and LCA Interactions within the cell. The generated models are based upon known and presumed interactions of (A) PCN and (B) LCA with nuclear receptors, and was generated using CellDesigner (v4.0.1; Systems Biology Institute, http://celldesigner.org/index.html). Each individual chemical or protein is identified as a species (s1.....sn), while interactions between species are identified as reactions (r1....rn). The generated models (closed squares) were able to reproduce *in vitro* (open squares) observed agonist-mediated suppression of PXR expression, and activation of CYP3A1, CYP24 and Fibrinogen B gene expression by agonist-activation of PXR, VDR and FXR respectively.

A notable feature of the transcript level measurements shown in Figure 2 is the down regulation of PXR transcript levels by both chemicals. Such data suggest that PXR-mediated transcriptional control may be subject to both feed-back and feed-forward control mechanisms, such that PXR activation both activates target gene expression and represses PXR expression. To understand the design principle behind this feed-back/feed-forward system we constructed two simple *in silico* models representing PCN interactions with PXR and its target gene CYP3A1 (Figure 6a), and LCA interactions with PXR, FXR and VDR, and their target genes CYP3A1, Fibrinogen B and CYP24 respectively (Figure 6b). These models were paramaterised with kinetic and quantitative data derived from published literature and the present study, and can replicate the effects of PCN and LCA on primary rat hepatocytes: Down regulation of PXR (PCN and LCA), plus the up regulation of the nuclear receptor target genes CYP3A1 (PCN and LCA), FGB (LCA only) and CYP24 (LCA only).

**Figure 7:**
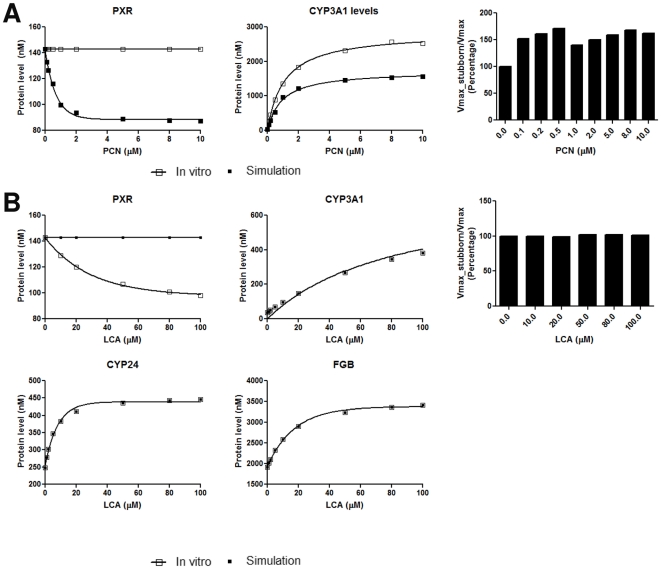
*In silico* simulation of the role of PXR autoregulation in the robustness of steroid biochemistry. The described *in silico* models were used for simulation as complete models (filled squares), or with the PXR autoregulatory feed-back loop disabled (stubborn PXR; open squares) following exposure to PCN (A) and LCA (B). Simulated protein levels of PXR, its target gene CYP3A1, and the Vmax against CYP3A substrates of the stubborn PXR system as a percentage of the complete model derived for a dose range of each agonist. In addition, protein levels of the VDR and FXR target genes CYP24 and Fibrinogen B, respectively, are also presented for LCA.

Following demonstration that the models were able to replicate the biological scenarios, we next examined the impact of PXR-mediated feed-back loop on these networks. The model was altered to create a ‘stubborn PXR’ system that lacks the negative feed-back loop, and produces a constant level of PXR regardless of PCN or LCA exposure (Figure 7). Under stubborn PXR conditions, no negative feed-back for PXR is present, resulting in effectively increased PXR levels; such effects result in a significant increase in CYP3A expression level for any given PCN exposure, being 162% of the level resulting from 10 µM PCN exposure under normal conditions (Figure 7a). In comparison, no alteration is seen in the levels of CYP3A following LCA exposure under stubborn PXR conditions (Figure 7b). Such a lack of effect may reflect the significantly lower affinity of LCA for PXR, with significant effects only being observed at much higher agonist concentrations. It is also important to note that the action of LCA as an agonist of VDR and FXR is not affected by the stubborn PXR condition, with no significant differences observed in the induction profiles for FGB and CYP24, which reflects the higher affinities of LCA for these two nuclear receptors.

To examine a potential biological rationale for this PXR negative feed-back loop we examined the impact of this attenuation of CYP3A expression on the metabolism of progesterone, testosterone and cortisol, all of which are CYP3A substrates. Simulation of the metabolism of these three steroids across a full dose response range of PCN or LCA demonstrated no impact on the Km for testosterone, progesterone or cortisol metabolism by CYP3A1 in the stubborn PXR system versus the full network. However, there is a significant impact on the absolute capacity (Vmax) for these processes following PCN exposure, due to the lack of decrease in concentration of activated PXR following PCN exposure in the stubborn PXR models. The relative Vmax in the stubborn PXR system, as a percentage of the Vmax in the whole network, was calculated and is shown in Figure 7: Interestingly, the resultant histogram for PCN treatment is biphasic, with the lowest point between maxima occurring at the Kd of PCN for PXR (∼1 µM).

**Figure 8:**
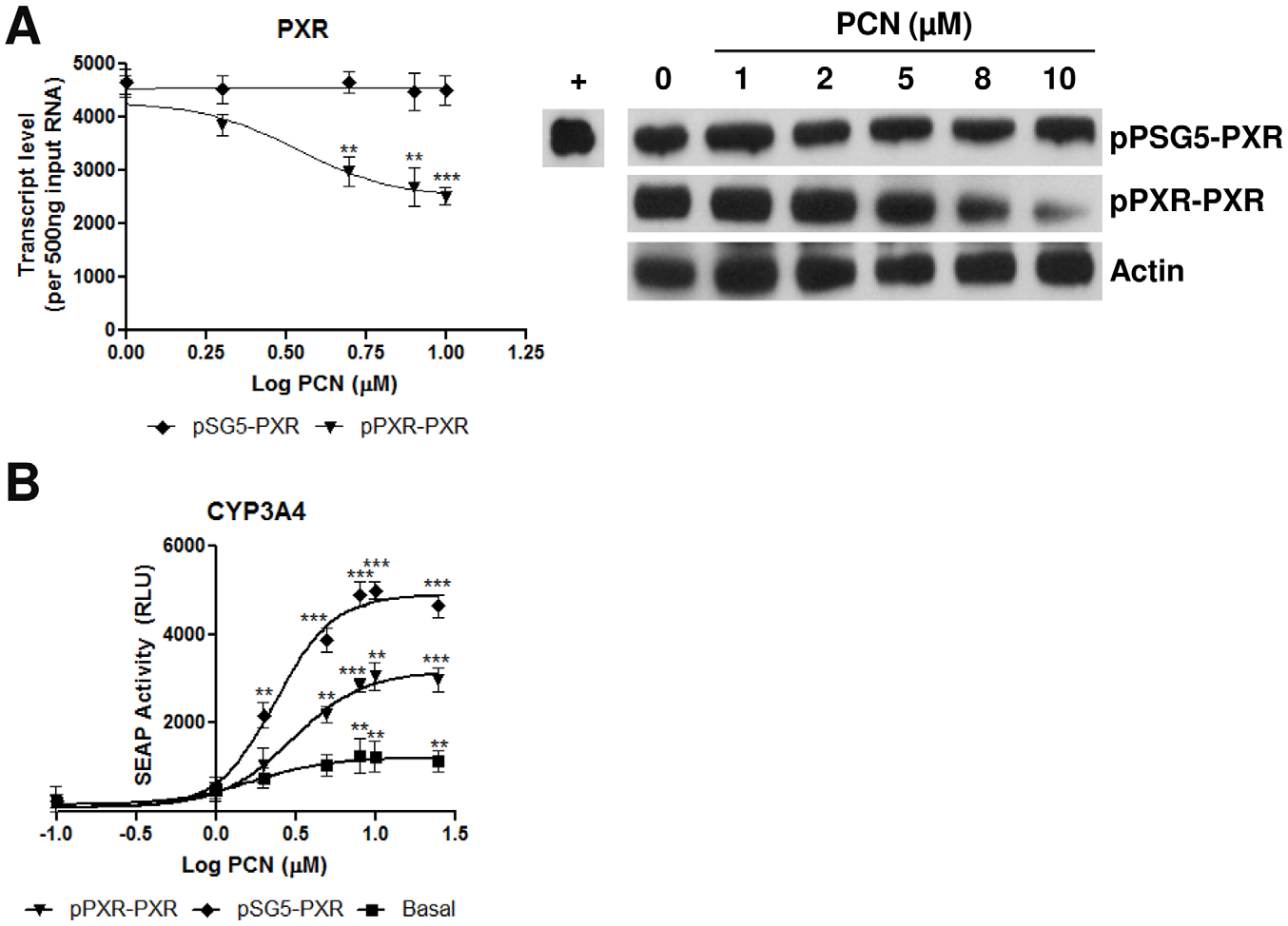
Negative feedback on PXR expression attenuates induction of CYP3A4 expression *in vitro*. Quadruplicate cultures of Huh7 cells were transfected with a CYP3A4 reporter gene alone, or with pSG5-PXR, pPXR-PXR. Following 24 hours incubation, cells were exposed to varying doses of PCN for 48 hours, as indicated, and then analysed: PXR transcript and protein levels are shown in (A), while reporter gene activity shown in (B). Statistical analysis against vehicle control was determined via one-way ANOVA; * = p<0.01, ** = p<0.05 and *** = p<0.001.

To determine if the attenuation of CYP3A gene expression predicted by the *in silico* model occurs in reality we next reproduced the *in silico* experiments in an *in vitro* setting: In addition, all components of the in vitro test system (cell line, target gene and PXR regulatory regions) were of human origin, allowing us to examine if the regulatory loop also functioned in the human context. The Huh7 human hepatoma cell line exhibits low level expression of PXR under normal conditions, and usually require addition of a PXR expression plasmid to elicit activation of reporter gene assays [30], primarily due to altered chromatin status [31]; they thus represent a near-PXR null system. By coupling the coding sequence for PXR to its cognate human proximal promoter (termed pPXR-PXR) it is possible to examine the role of transcriptional level control of PXR expression on the activation of downstream target genes such as CYP3A4. It should be noted that in silico analysis of the human and rat PXR proximal promoter regions demonstrates a high conservation of putative transcription factor binding sites, with conserved binding sites for numerous nuclear receptors including PXR, VDR and FXR (data not shown).Regulation of pPXR-PXR by PXR ligands could be demonstrated, with 10 µM PCN eliciting a decrease in PXR transcript and protein levels to 58% and 41% respectively, compared to vehicle control; by comparison, no PCN-mediated alteration in PXR expression levels was observed using the pSG5-PXR plasmid, where PXR expression is under control of a heterologous promoter, was observed at either transcript or protein level (Figure 8a).

We next examined the impact of this on PXR target gene expression; Huh7 cells were co-transfected with a CYP3A4 SEAP reporter gene, plus either pSG5-PXR or pPXR-PXR. Cells were exposed to a concentration curve of PCN between 0.1 µM and 25 µM for 48 hours and then SEAP activity measured. While PCN is a better agonist of rodent PXR compared to human PXR, it is still an effective activator of human PXR, as previously reported. [30]. In the current study, co-transfection with pSG5-PXR results in an increased CYP3A4 reporter gene expression compared to basal PXR levels, with a maximal fold increase of 49-fold over vehicle alone and an EC50 = 2.4±1.1 µM (Figure 8b). Co-transfection with the pPXR-PXR construct also resulted in a significant increase in CYP3A4 reporter gene expression following PCN exposure, with a maximal 31.6-fold increase and an EC50 = 3±1.1 µM, but this was significantly lower than the increase observed with pSG5-PXR. Such data is consistent with a scenario where PCN-mediated negative regulation of PXR expression is used to attenuate CYP3A gene expression.

## Discussion

Biological response pathways to chemical challenge must exhibit both sensitivity and robustness: The body must be able to respond rapidly to either challenge by xenobiotics or changes in endogenous chemical levels that could result in pathophysiology (sensitive), while at the same time not allowing this sensitivity to significantly impact on the endogenous metabolic processes that utilize the same enzymes (robustness). It is becoming clear that for this to occur, the body must utilize a network of interactions, allowing the most efficient response to any given stimulus [3], [32]. In the current study we have examined how feed-forward and feed-back loops are used to control the response to both endogenous chemicals and xenobiotics, delivering the required sensitivity and robustness within the network.

The activation of rat transcriptome by the exogenous chemical PCN has been well studied, both by ourselves and others, demonstrating a PXR-mediated activation of catabolic and transport pathways designed to eliminate the stimulating chemical [32], [33], [34]. Such a response is logical, given that the chemical is external by nature and, hence, the body will move to reduce its concentration to zero through up-regulation of metabolic enzymes and drug transporters. By comparison, the transcriptomic response to bile acids is more complicated: At low concentrations, negative feed-back on bile formation may be sufficient to prevent a build-up to toxic concentrations. However, as the level of toxic bile acids approaches that eliciting cholestasis, the body requires a more frank response, including both an increase in catabolic processes and protective pathways. This differential response is demonstrated by the PCA for transcriptome responses to the two ligands, showing first overlay and then divergence of the trajectories. Given the relative EC50s for activation of VDR, FXR and PXR by the two chemicals, at the lower exposure doses it is logical that LCA target gene-activation is predominantly via VDR, while PCN activation is through PXR. Given this disparate nuclear receptor activation it is perhaps surprising that both agents activate a highly correlated gene expression profile. However, it is well established that VDR can activate expression of target genes traditionally associated with PXR through response element sharing [32], [35], and this could explain the overlapping PCA PC1 trajectories, which are primarily determined by activation of metabolic genes. The activation of a general metabolic response, combined with the activation of VDR- and FXR-specific target genes, should be sufficient to cause a return to homeostasis at these lower concentrations of LCA. However, if the concentration of LCA continues to rise, the percentage activation of PXR will also increase, leading to increased metabolic activation of, for example, CYP3A1, plus the activation of specific target genes geared towards meeting the response to cholestatic injury.

A novel finding of the current work is the activation of expression for the metallothionein 1a and 2 genes by LCA. Such a response can almost certainly be attributed to the oxidative stress caused during cholestasis [36]. FXR knockout mice show an increased susceptibility to bile acid-induced ROS damage, suggesting that activation of these genes is, at least in part, under the control of FXR [37]. However, in the current study, activation of these protective gene products occurs at higher LCA concentrations, when PXR will be significantly activated. Zilliacus and colleagues have previously suggested that MTs can be regulated by PXR [38], and the data presented herein is consistent with such a scenario. We would therefore postulate that PXR activation by LCA becomes significant as LCA concentration approaches that eliciting pathophysiology, resulting in the production of protective mechanisms to reduce the associated oxidative stress, namely increased MT1a and 2 and SOD1 transcript levels.

Taken together these findings would suggest that the regulation of bile acid homeostasis in rats is through the action of the PXR, VDR and FXR nuclear receptors working as a partnership of complimentary pathways, adjusting the pattern of gene expression for extent of divergence from bile acid homeostasis, aiming to ensure healthy physiology. Such a interaction is demonstrated well in knockout mice, with FXR and PXR individual gene knockouts showing a reduced, but still strong, response to bile acid loading [39], whereas the PXR/FXR double knockout mice show a poor response to bile loading [40]. This poor response indicates that while CAR, LXR and VDR are all implicated in small areas of bile acid biosynthesis, FXR and PXR are the dominant receptors responsible for control in this system [40].

As stated above, one level at which refinement of biological response to chemical stimulus can be achieved is through the interaction of nuclear receptors signalling, via auto- or trans-regulatory loops, or through competition for ligands and/or response elements. Herein, we demonstrate that PXR is under control of a feed-back loop, with both PCN and LCA reducing PXR transcript levels to approximately 50% of that seen with no agonist present; however, activation of this feed-back loop only occurs at pathophysiological concentrations for LCA. Such data is consistent with previous observations that over-expression of PXR *in vitro* results in a decrease in PXR reporter gene activity [21], with these latter experiments being supportive for such regulation occurring at the transcriptional level. However, Maglich et al. observed a 2.7-fold increase in PXR transcript levels in mice following exposure to PCN, which was ablated in a PXR knockout animal [41]. Whereas both findings are consistent with a PXR-mediated regulation of PXR transcript levels, they differ in the directionality of this change. The inconsistency of these findings may be a product of the differing test systems, primary rat hepatocytes versus mouse *in vivo*, or the exposure period, 48 hours versus 28 hours. It should be noted that the findings presented herein are supported by changes at the protein level as well, demonstrating the observed transcriptome level effects are translated to the proteome. Whereas such data is consistent with the presented hypothesis, it should be noted that alternate hypothesis may be envisaged; for example, ligand stimulation might increase targeting of the activated receptor molecule to the proteosome, as seen with other nuclear receptors [42]. Such an effect would also decrease protein levels, but not via a transcriptional route. Given the transcriptional-level effects observed both herein and by Maglich et al., it is likely that such a mechanism would be complimentary, not alternate, to any transcriptional level control; indeed, the altered protein levels resulting from degradation rate increases might actually be the driver for an alteration in transcription rates, but further work would be required to evaluate this. In addition, it should be noted that no measurement of potential post-translational modifications have been undertaken, which could further modulate the activity of a liganded nuclear receptor [43].

The decrease in PXR protein levels observed herein could serve two important processes: First, ensuring that the levels of free agonist remain high, potentially increasing interaction of the agonist with other nuclear receptors. Second, a reduction in the total amount of PXR protein will reduce the total amount of activated PXR protein, as this is a function of receptor occupancy; such a reduction in activated PXR could impact upon the expression level of downstream target genes, such as CYP3A1, as proposed herein. This would attenuate PXR target gene induction at higher agonist concentrations, preventing an over-response of the biological system to hyper-stimulation. Given that order of nuclear receptor activation by LCA is VDR≈FXR>>PXR, then the first scenario is unlikely to be important, as at concentrations of LCA where PXR negative feed-back becomes significant, receptor occupancy for VDR and FXR is already approaching maximal. We therefore postulate that the second scenario is more probable, whereby PXR negative feed-back is utilized to attenuate PXR-mediated activation of target genes at higher agonist concentrations. Such a hypothesis is supported by the *in vitro* data demonstrating attenuation of CYP3A induction by PCN, and by the *in silico* simulation demonstrating significantly higher turnover rates for three endogenous CYP3A substrates, testosterone, progesterone and cortisol, when the feed-back loop is removed. This data is highly supportive of the notion that the PXR feed-back loop is required for the robustness of steroid homeostasis in the case of steroids that are CYP3A substrates, and is logical with regard to cellular energetic position, as it is far more efficient to attenuate degradation of these steroids rather than increase de novo synthesis to compensate for increased degradation. Interestingly, this attenuation appears to be biphasic, with maximal attenuation occurring at the Kd for the agonist, although the rationale for this is unclear. It should also be noted that this autoregulatory loop may not always be activated, and indeed we have preliminary evidence to suggest that this is often the case with endogenous ligands, with activation of PXR by some glucocorticoids, progestins and androgens not appearing to activate this feed-back loop (unpublished data). This minimal/absent use of the PXR feed-back loop by endogenous agonists may be logical, as concentrations of endogenous ligands high enough to significantly activate PXR would only occur in pathophysiological conditions when it would be important to return to homeostasis as soon as possible and not limit the rate of CYP3A1-mediated degradation. It is thus tantalizing to suggest that PXR can sense those ligands that may impact upon endogenous metabolism and activate the autoregulatory loop, while disengage the loop for endogenous chemicals to allow a more efficient return to homeostasis. Such a bipartite response could reflect the recruitment of differing co-regulators to the agonist-PXR complex, either allowing or preventing repression of PXR gene expression. Regulation of PXR by other nuclear receptors has already been established [12], [14], although the molecular mechanisms for such interactions are not always clear, with the exception of PPARα [14]. Glucocorticoid-mediated induction of PXR expression is likely to be mediated through the glucocorticoid receptor, with a GRE present within the PXR proximal promoter [14]. However, no PXRE exists within the PXR proximal promoter; instead it is tempting to postulate that any such regulation would occur through interaction with the VDREs present within this regulatory region, as PXR has already been shown to be able to function in this manner [44]: However, further work is required to confirm such speculation.

In summary, the current study has demonstrated that PXR not only acts as a xenosensor in the classical sense, but also potentially acts as an ‘endosensor’, allowing it to coordinate responses to chemical exposure in a tailored fashion dependent upon the nature of the chemical challenge and its likely impact on body homeostasis.

## Materials and Methods

### Ethics Statement

All animal experiments described herein were undertaken in full compliance with the relevant sections of the UK Government Scientific Procedures Act (1986) for the use of animals in experimentation. Termination of rats for primary hepatocyte preparation was covered by the general project licence 40/2892 held by AstraZeneca Safety Assessment, UK, which was approved by the UK Government Home Office Animal Procedures Committee under A(SP)A 1986.

### Chemicals

PCN and LCA were of cell culture grade and purchased from Sigma Chemical Co. (St. Louis, MO). Unless otherwise stated all other chemicals were of molecular biology grade and obtained from Sigma Chemical Co. (St. Louis, MO).

### Cell Culture

Primary rat hepatocytes were isolated from AZ Sprague-Dawley rats and grown in sandwich culture system using RPMI medium, supplemented with 10% foetal bovine serum, 50 mg/ml pen/strep and 1×10^−7^ M human insulin. Cells were initially plated at a density of 1×10^5^ cells/cm^2^ for 4 days; at this point functioning bile canaliculi were formed, confirmed using the ABCC2-specific substrate carboxydichlorofluroscein, which will only be transported in correctly polarised cells [26] (data not shown). Following formation of functioning cannaliculi, cells were dosed daily to varying concentrations of PCN (1, 2, 5, 8, 10 µM), LCA (10, 20, 50, 80, 100 µM) or vehicle control (0.1% DMSO). Following 48 hours of exposure, cells were lyzed and RNA isolated and purified using the RNAqueous-4PCR kit (Ambion, Austin, TX), and quantified using a Nanodrop Agilent 2100 Bioanalyser. Compound mediated toxicity was assessed by LDH assay, and was shown to be non-significant at all concentrations tested (data not shown).

The Huh7 human hepatocellular carcinoma cell line [45] was a kind gift from Dr Steve Hood (GSK, Ware, UK). All cells were routinely cultured in 75 cm^2^ vented tissue culture flasks (Nunc, UK) using minimal essential medium with Earle's salts supplemented with 1% non-essential amino acids, 2 mM L-glutamine, 100 U/ml penicillin, 100 µg/ml streptomycin and 10% foetal bovine serum. In order to maintain phenotypic consistency, Huh7 cells were only used for three weeks (approximately 5 passages) following recovery from liquid nitrogen.

### DNA Microarray and Analysis

Total RNA was processed and labelled for microarray analysis using the One cycle target labelling and control reagents kit (Affymetrix, Santa Clara, CA), and used to interrogate the Affymetrix rat 230 v2 GeneChip set, representing over 30,000 transcripts including approximately 28,000 well substantiated rat genes.

The raw data generated from the microarray was first normalised using the Affymetrix algorithm, which allows reliable comparison of multiple arrays through minimising differences of non-biological origin. All data was produced in a MIAME-compliant format [46], with the normalised output from the microarray presented as File S1.

Next, the SAM (Significance Analysis of Microarrays) package (http://www-stat.stanford.edu/~tibs/SAM/) was used to identify significantly altered gene expressions, and generate false discovery rate (FDR) values for the analysis. The SAM package offers the advantage over other microarray analysis tools in that it does not presume equal variance or independence of genes (or both), scenarios that are often violated in biological systems [47]. Heatmaps from the SAM output were generated using Cluster 3.0 [48] and visualised using Java TreeView [49], while Principle Component Analysis was undertaken using MVSP (Kovach Computing Service, Anglesey, UK).

To examine the potential impact on biological pathways of the identified gene expression changes the DAVID software suite (http://david.abcc.ncifcrf.gov/home.jsp) was used to undertake functional annotation clustering, whereby GO identifiers that are statistically over-represented are clustered according to their biological functions [50]. The full output from the microarray experiment has been deposited with public GEO database (www.ncbi.nlm.nih.gov/geo).

### Transcript level measurement

Specific primers and TAMRA/FAM dual labelled probe sets were designed against all target genes using the Primer Express software (Applied Biosystems, Warrington, UK) and were purchased from Eurofins MWG (Wolverhampton, UK): Sequences for all probe/primer sets are presented as File S2.

Total RNA was treated with RNase-free DNase (Promega, Southampton, UK) to remove genomic contamination. Reverse transcription was primed with random hexamers and carried out by Superscript II (Invitrogen) as per the manufacturer's instructions. To ensure that DNase treated samples were free from genomic contamination an RT- control (lacking enzyme) was carried out for every RNA sample. cDNA amplified using TaqMan Universal PCR Master Mix with 400 nM primers and 200 nM fluorogenic probe in a total reaction volume of 25 µl: In general, cDNA generated from 50 ng input total RNA was used per reactions, with the exception of 18 s detection, where cDNA derived from 50 pg input total RNA was used. Quantitative polymerase chain reaction (Q-PCR) reactions were run on the ABI7000 SDS instrument and quantitation was carried out using the ABI proprietary software against a standard curve generated from human genomic DNA (Promega), and normalised against 18 s rRNA expression levels.

### Protein level measurement

Primary rat hepatocytes (Invitrogen) or Huh7 cells were seeded at 2.4×10^5^ cells/cm^2^ in 25 cm^2^ flasks and treated as described above. Following 48 hours of exposure to vehicle, PCN or LCA total protein was extracted in RIPA buffer (1xPBS, 1% Nonidet P40, 0.5% sodium deoxycholate, 0.1% SDS, protease inhibitor cocktail).

Total protein extracts (10 µg per lane) were resolved on 12% SDS-polyacrylamide gels and then transferred electrophoretically to Hybond ECL nitrocellulose membranes (Amersham Biosciences, Little Chalfont, Bucks, UK). Membranes were blocked (1 hour) in 5% fat free dried milk and then probed with primary antibodies against human PXR (SAB2101636, 1∶350; Sigma-Aldrich) or α-actin (sc1616, 1∶500; Autogen Bioclear), followed by anti-rabbit IgG (sc2030, 1∶10000; Autogen Bioclear) or anti-goat IgG (sc 2020, 1∶20000; Autogen Bioclear) respectively. Bound antibodies were visualised using enhanced chemiluminescence reagents according to the manufactures instructions (Amersham Biosciences).

### Reporter gene assay

Huh7 cells were seeded into 96-well plates (Nunc International, Leicestershire, UK) at a concentration of 10,000 cells/well and incubated at 37°C for 24 hrs in a humidified container for attachment. FuGENE 6-mediated DNA co-transfections, using CYP3A4-XREM reporter gene construct [30], plus an expression plasmid for PXR, either under the control of a minimal promoter or the cognate human 2.2 kb PXR proximal promoter. Following 24 hours incubation, cells were exposed to vehicle or drug and allowed to proceed for 48 hours, and secretory alkaline phosphatase (SEAP) activity measured: Briefly, aliquots of cell culture medium (25 µl/well) were transferred into 96-well optiplates (Canberra Packard, UK), endogenous alkaline phosphatase activity deactivated by heat-treatment and then SEAP activity assayed using the AURORA system (ICN, Thame, UK), according to the manufacturer's protocol. Chemiluminescent output was measured using a LumiCount automated plate reader (Canberra Packard, UK).

SEAP activity following 48 hours culture was calculated for both reporter constructs and blank, control, plasmid, and a fold induction relative to vehicle control calculated.

### *In silico* Modelling

*In silico* models were generated using CellDesigner (v4.0.1; Systems Biology Institute, http://celldesigner.org/index.html), a graphical front-end for creating process diagrams of biochemical networks in systems Biology Markup Language (SBML; [51]). Each individual chemical or protein is identified as a species (s1.....sn), while interactions between species are identified as reactions (r1....rn). For each reaction, a kinetic term is included, detailing the mathematics underlying the interaction of the species: A full diagrammatic representation, plus paramaterisation values for each model are presented as File S3.

## Supporting Information

File S1 **Raw Gene Array Output.** Quadruplicate cultures of primary rat hepatocytes were exposed to PCN or LCA at the indicated concentrations for 48 hours. RNA was extracted and gene expression monitored using Affymetrix rat 230 v2 microarrays. Fluorescent output for each probe on each array is presented, following normalisation using the Affymetrix algorithm.
(XLS)

File S2 **TaqMan Probe sets.** Specific primers, plus TAMRA/FAM dual labelled probes, were designed against the indicated RefSeq for each target gene using the Primer Express software (Applied Biosystems, Warrington, UK). Probe primer sets were designed to cross an intron-exon boundary, with design parameters set as defaults for the software.
(DOC)

File S3 **Model Parameters.** *In silico* models for both PCN- and LCA-response networks were generated using CellDesigner (v4.0.1), represented in the Systems Biology Graphical Notation (SBGN) images. Expression levels of individual species, kinetic descriptions of reactions, plus initial state values for each network are also provided.
(DOC)
