# Supplementary material for: A PXR-Mediated Negative Feedback Loop Attenuates the Expression of CYP3A in Response to the PXR Agonist Pregnenalone-16α-Carbonitrile
Source: PLoS One. 2011 Feb 2;6(2):e16703. doi: 10.1371/journal.pone.0016703 (PMC3032768; doi:10.1371/journal.pone.0016703)
Supplement: File S2 — TaqMan Probe sets. Specific primers, plus TAMRA/FAM dual labelled probes, were designed against the indicated RefSeq for each target gene using the Primer Express software (Applied Biosystems, Warrington, UK). Probe primer sets were designed to cross an intron-exon boundary, with design parameters set as defaults for the software. (DOC) [file pone.0016703.s002.doc]

| **Target Gene** | **RefSeq** | **Forward Primer** | **Reverse Primer** | **Probe** |
| --- | --- | --- | --- | --- |
| CYP3A1 | NM_013105 | TGCTGGATATGAACCCACCA | TGTATCTGGGTGAGTGGCCA | CAGCACACTTTCCTTTGTCCTGCATTCC |
| ABCC2 | NM_012833 | GGCAATGGTGTGTACGAAAG | TTGGACTTGCTGTCCTTCAG | TCCTCTCGGTCTTATGCGGCG |
| GSTA2 | NM_017013 | TGAGAAGTTTATACAGAGTCCAGAAG | TGGCGATGTAGTTGAGAATGG | AGATTGACGGGATGAAGCTGGCA |
| CYP24A1 | NM_201635 | AGCGACTGGAGATCAAACCT | TGTCCTTCCAGGATCATCAA | CGCGACCACAGGAACGAAGC |
| KLK6 | NM_019175 | AGAGGGACTTACAGGTCGGA | CCACCTATCACAGGCAGAGA | TGTGGAGCACCCTGCCTTGC |
| Fibrinogen B | NM_020071 | TGTGAACGATAATATCCCCCTTAATC | TGCATTTTGCTTCTCAGGTCC | TCGTGTGCTCCGCTCAATCCTGG |
| UGT2B4 | NM_001004271 | CCACCCAATATCGCCAAATG | TTGACCCCAGAGAAAACACC | TCCACTGCAAACCTGCTAAACCCT |
| PXR | NM_052980 | TCTTCTCCCCAGATCGCC | CAAATCGCTCCTGCAGCT | TCTACCACGCTACGTTGAACCACGCC |
| SHP | NM_057133 | TTCAACCCAGATGTGCCA | GACTTCACACAATGCCCAGT | CCTCCATGCCTCCTGCCACA |
| FXR | NM_021745 | GCAGACCTGTTGGAAGAAAG | AGAGCGTACTCTTCCTGGGT | CGAAAGAGCGGCATCTCCGA |
| VDR | NM_017058 | GGATTCAGGGATCTCACCTC | ATGGTGAAAGACTGGTTGGA | TGATCACCTCAATGGCGCTTGA |
| GSTA4 | NM_001106840 | CAATCACAGCAGCAGCTACA | ATTGGCATTTGTCATTGTGG | TCCTGAGAGCTCTATCTTGCCTCTGGA |
| GSTM2 | NM_177426 | GAGAACCAGGCTATGGACAC | TCTCTCAAAGTCAGGGCTGT | CCGCCTACAGTTGGCCATGG |
| CYP2C70 | NM_138512 | CTCGTAAGACAACGCAGGAT | CTCTGGGTTGGGAAATTCTT | CATTCCCAAGGGCACAAGCG |
| UGT2B5 | NM_153314 | TGCCCAAGGATATGGAAGAT | ATTGGGTCCTAAGGTTGCTG | CAATGCAATTGCATGGGCCC |
| MT1a | NM_138826 | AAAGGTGCCTCGGACAAG | TGCCTGAAGTGACGAACAGTGCT | TCACATGCTCGGTAGAAAACG |
| MT2 | NM_001137564 | TCACGCTCCTAGAACTCTGC | GAGCAGGATCCATCTGTGG | TCCAACTGCCGCCTCCATTC |
| MT3 | NM_053968 | TGCCTGGAGGAACTAAGCTA | CAGGAACCACCAGTAGGACA | CATATCCAGGCCAGCAGCCG |
| GPx1 | NM_030826 | ATGAATGATCTGCAGAAGCG | TCTTGCCATTCTCCTGATGT | TGCTCGGTTTCCCGTGCAAT |
| GPX2 | NM_183403 | GGACTACACCCAGCTCAATG | ACAGTTCTCCTGATGTCCGA | CGCTTTCCCAGGCGCCTAGT |
| GPX3 | NM_022525 | AGCCAGCTACTGAGGTCTGA | GAATGGGCCAAGTTCTTCTT | CAGACCAATACCTTGAACTGAATGCA |
| SOD1 | NM_017050 | GAAGAGAGGCATGTTGGAGA | CACGATCTTCAATGGACACA | TTGGCCACACCGTCCTTTCC |
| SOD2 | NM_017051 | GGCCATATCAATCACAGCAT | AGCAACTCTCCTTTGGGTTC | CCACCACCCTTAGGGCTCAGG |
| SOD3 | NM_012880 | CTTGTCAGGTGTGGAACCTC | TCTCCGGTATCTGACATGGT | TGGCCTGTGGCTCTGTCACC |

Notes:

1. All probe primer sets were designed against the indicated RefSeq
2. All probe-primer sets were designed to cross and intron-exon boundary
3. All probes were labelled with Fam-TAMRA
