# Supplementary material for: A PXR-Mediated Negative Feedback Loop Attenuates the Expression of CYP3A in Response to the PXR Agonist Pregnenalone-16α-Carbonitrile
Source: PLoS One. 2011 Feb 2;6(2):e16703. doi: 10.1371/journal.pone.0016703 (PMC3032768; doi:10.1371/journal.pone.0016703)
Supplement: File S3 — Model Parameters. In silico models for both PCN- and LCA-response networks were generated using CellDesigner (v4.0.1), represented in the Systems Biology Graphical Notation (SBGN) images. Expression levels of individual species, kinetic descriptions of reactions, plus initial state values for each network are also provided. (DOC) [file pone.0016703.s003.doc]

**SBGN representation of PCN-PXR interaction model**


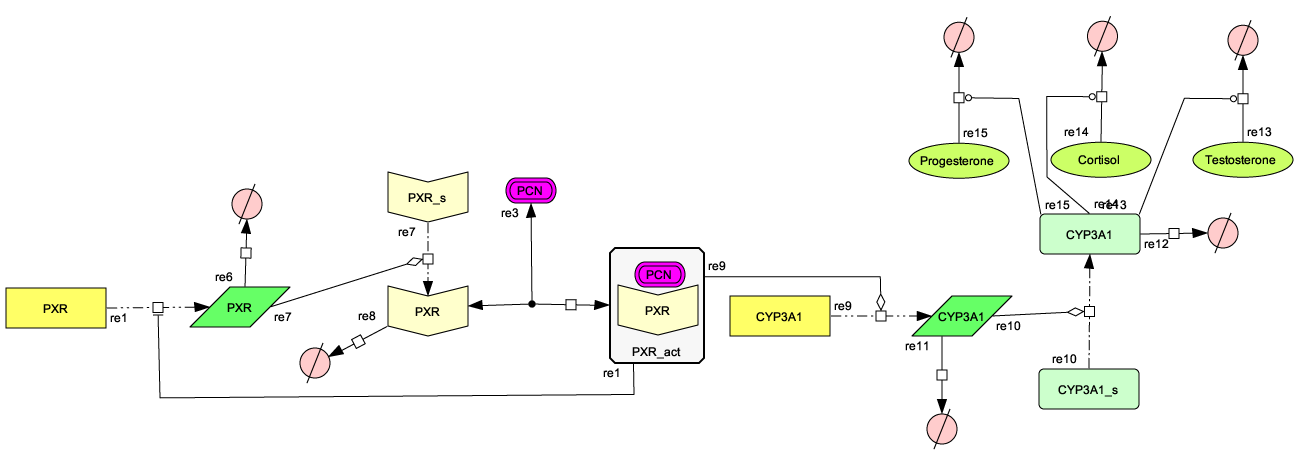


**Parameters for PCN-PXR model**

| **Reaction Parameters** | | | |
| --- | --- | --- | --- |
| **Reaction** | **ID** | **Description** | **Value** |
| re1 |  | PXR Gene Expression  *k1·(1+PXRact/ki)+k2 (t)* [nM·min^-1^] |  |
|  | k1 | Repressible synthesis rate | 0.25 min^-1^ |
|  | k2 | Basal synthesis rate | 0.1 min^-1^ |
|  | ki | ki for PXRact on PXR gene expression | 66 nM |
| re3 |  | PXR-PCN Binding  *kon·PCN·PXRprot – koff (t)* [nM·min^-1^] |  |
|  | kon | kon | 0.06 nM·min^-1^ |
|  | koff | Koff | 60 min^-1^ |
| re6 |  | PXR RNA: Degradation rate  *k1·PXR RNA (t)* [nM·min^-1^] |  |
|  | k1 | k1 | 0.35 min^-1^ |
| re7 |  | PXR Protein: Synthesis rate  *k1·PXR RNA (t)* [nM·min^-1^] |  |
|  | k1 | k1 | 10 min^-1^ |
| re8 |  | PXR Protein: Degradation rate  *k1·PXR Protein (t)* [nM·min^-1^] |  |
|  | k1 | k1 | 0.07 min^-1^ |
| re9 |  | CYP3A1 Gene Expression  *k1·(PXRact/(PXRact+PXR))·kact+k2 (t)* [nM·min^-1^] |  |
|  | k1 | Activatable Synthesis rate | 0.3 min^-1^ |
|  | k2 | Basal Synthesis rate | 0.05 min^-1^ |
|  | kact | kact for PXR effect on synthesis | 7.8 |
| re10 |  | CYP3A1 Protein: synthesis rate  *k1·CYP3A1 RNA (t)* [nM·min^-1^] |  |
|  | k1 | k1 | 10 min^-1^ |
| re11 |  | CYP3A1 RNA: Degradation rate  *k1·CYP3A1 Protein (t)* [nM·min^-1^] |  |
|  | k1 | k1 | 0.19 min^-1^ |
| re12 |  | CYP3A1 Protein: Degradation rate  *k1·CYP3A1 Protein (t)* [nM·min^-1^] |  |
|  | k1 | k1 | 0.07 min^-1^ |
| re13 |  | Testosterone Metabolism by CYP3A1  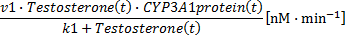 |  |
|  | v1 | Vmax | 33 nM·min^-1^ |
|  | k1 | Km | 33000 nM |
| re14 |  | Cortisol Metabolism by CYP3A1  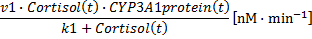 |  |
|  | v1 | Vmax | 0.067 nM·min^-1^ |
|  | k1 | Km | 10000 nM |
| re15 |  | Progesterone Metabolism by CYP3A1  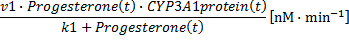 |  |
|  | v1 | Vmax | 33 nM·min^-1^ |
|  | k1 | Km | 80000 nM |

**Initial Conditions** for PCN-PXR model

| **Parameter** | **Value** |
| --- | --- |
| PXR Gene | 1 * |
| PXR RNA | 1 nM |
| PXR Protein | 143 nM |
| PXRact | 0 nM |
| PXR_s | 1 * |
| CYP3A1 Gene | 1* |
| CYP3A1 RNA | 0.263 nM |
| CYP3A1 Protein | 37.6 nM |
| CYP3A1_s | 1* |
| Progesterone | 30 nM† |
| Cortisol | 220 nM† |
| Testosterone | 15 nM† |

* Values marked with an asterix are fixed boundary conditions within the model

†initial concentrations for steroids are based upon mean circulating plasma concentrations

**SBGN representation of LCA-PXR-FXR-VDR interaction model**

**Parameters for PCN-PXR-FXR-VDR model**

| **Reaction Parameters** | | | |
| --- | --- | --- | --- |
| **Reaction** | **ID** | **Description** | **Value** |
| re1 |  | PXR Gene Expression  *k1·(1+PXRact/ki)+k2 (t)* [nM·min^-1^] |  |
|  | k1 | Repressible synthesis rate | 0.25 min^-1^ |
|  | k2 | Basal synthesis rate | 0.1 min^-1^ |
|  | ki | ki for PXRact on PXR gene expression | 120 nM |
| re3 |  | PXR-LCA Binding  *kon·LCA·PXRprot – koff (t)* [nM·min^-1^] |  |
|  | kon | kon | 0.06 nM·min^-1^ |
|  | koff | Koff | 5700 min^-1^ |
| re6 |  | PXR RNA: Degradation rate  *k1·PXR RNA (t)* [nM·min^-1^] |  |
|  | k1 | k1 | 0.35 min^-1^ |
| re7 |  | PXR Protein: Synthesis rate  *k1·PXR RNA (t)* [nM·min^-1^] |  |
|  | k1 | k1 | 10 min^-1^ |
| re8 |  | PXR Protein: Degradation rate  *k1·PXR Protein (t)* [nM·min^-1^] |  |
|  | k1 | k1 | 0.07 min^-1^ |
| re9 |  | CYP3A1 Gene Expression  *k1·(PXRact/(PXRact+PXR))·kact+k2 (t)* [nM·min^-1^] |  |
|  | k1 | Activatable Synthesis rate | 0.3 min^-1^ |
|  | k2 | Basal Synthesis rate | 0.05 min^-1^ |
|  | kact | kact for PXR effect on synthesis | 3 |
| re10 |  | CYP3A1 Protein: synthesis rate  *k1·CYP3A1 RNA (t)* [nM·min^-1^] |  |
|  | k1 | k1 | 10 min^-1^ |
| re11 |  | CYP3A1 RNA: Degradation rate  *k1·CYP3A1 Protein (t)* [nM·min^-1^] |  |
|  | k1 | k1 | 0.25 min^-1^ |
| re12 |  | CYP3A1 Protein: Degradation rate  *k1·CYP3A1 Protein (t)* [nM·min^-1^] |  |
|  | k1 | k1 | 0.07 min^-1^ |
| re13 |  | FXR-LCA Binding  *kon·LCA·FXRprot – koff (t)* [nM·min^-1^] |  |
|  | kon | kon | 0.06 nM·min^-1^ |
|  | koff | Koff | 840 min^-1^ |
| re14 |  | VDR-LCA Binding  *kon·LCA·VDRprot – koff (t)* [nM·min^-1^] |  |
|  | kon | kon | 0.06 nM·min^-1^ |
|  | koff | Koff | 300 min^-1^ |
| re15 |  | CYP24 Gene Expression  *k1·(VDRact/(VDRact+VDR))·kact+k2 (t)* [nM·min^-1^] |  |
|  | k1 | Activatable Synthesis rate | 0.1 min^-1^ |
|  | k2 | Basal Synthesis rate | 0.2 min^-1^ |
|  | kact | kact for PXR effect on synthesis | 0.005 |
| re17 |  | CYP24 RNA: Degradation rate  *k1·CYP24 Protein (t)* [nM·min^-1^] |  |
|  | k1 | k1 | 0.0303 min^-1^ |
| re18 |  | CYP24 Protein: synthesis rate  *k1·CYP24 RNA (t)* [nM·min^-1^] |  |
|  | k1 | k1 | 10 min^-1^ |
| re19 |  | CYP24 Protein: Degradation rate  *k1·CYP24 Protein (t)* [nM·min^-1^] |  |
|  | k1 | k1 | 0.266 min^-1^ |
| re20 |  | FGB Gene Expression  *k1·(FXRact/(FXRact+FXR))·kact+k2 (t)* [nM·min^-1^] |  |
|  | k1 | Activatable Synthesis rate | 0.1 min^-1^ |
|  | k2 | Basal Synthesis rate | 0.2 min^-1^ |
|  | kact | kact for PXR effect on synthesis | 1.8 |
| re21 |  | FGB RNA: Degradation rate  *k1·FGB RNA (t)* [nM·min^-1^] |  |
|  | k1 | k1 | 0.0274 min^-1^ |
| re22 |  | FGB Protein: synthesis rate  *k1·FGB RNA (t)* [nM·min^-1^] |  |
|  | k1 | k1 | 10 min^-1^ |
| re23 |  | FGB Protein: Degradation rate  *k1·FGB Protein (t)* [nM·min^-1^] |  |
|  | k1 | k1 | 0.005235 min^-1^ |
| re24 |  | Testosterone Metabolism by CYP3A1  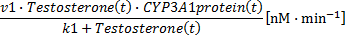 |  |
|  | v1 | Vmax | 33 nM·min^-1^ |
|  | k1 | Km | 33000 nM |
| re25 |  | Cortisol Metabolism by CYP3A1  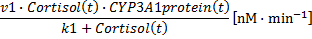 |  |
|  | v1 | Vmax | 0.067 nM·min^-1^ |
|  | k1 | Km | 10000 nM |
| re26 |  | Progesterone Metabolism by CYP3A1  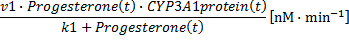 |  |
|  | v1 | Vmax | 33 nM·min^-1^ |
|  | k1 | Km | 80000 nM |

**Initial Conditions for PCN-PXR-FXR-VDR model**

| **Parameter** | **Value** |
| --- | --- |
| PXR Gene | 1 * |
| PXR RNA | 1 nM |
| PXR Protein | 143 nM |
| PXRact | 0 nM |
| PXR_s | 1 * |
| VDR Gene | 1* |
| VDR Protein | 334 nM |
| VDRact | 0 nM |
| FXR Gene | 1* |
| FXR Protein | 1192 nM |
| FXRact | 0 nM |
| CYP3A1 Gene | 1* |
| CYP3A1 RNA | 0.263 nM |
| CYP3A1 Protein | 37.6 nM |
| CYP3A1_s | 1* |
| CYP24 Gene | 1* |
| CYP24 RNA | 6.6 nM |
| CYP25 Protein | 248 nM |
| CYP24_s | 1* |
| FGB Gene | 1* |
| FGB RNA | 7.3 nM |
| FGB Protein | 1910 nM |
| FGB_s | 1* |
| Progesterone | 30 nM† |
| Cortisol | 220 nM† |
| Testosterone | 15 nM† |

* Values marked with an asterix are fixed boundary conditions within the model

†initial concentrations for steroids are based upon mean circulating plasma concentrations
